# Promising Anti-Inflammatory Tools: Biomedical Efficacy of Lipoxins and Their Synthetic Pathways

**DOI:** 10.3390/ijms241713282

**Published:** 2023-08-27

**Authors:** Junxi Chi, Jiahao Cheng, Shang Wang, Cheng Li, Ming Chen

**Affiliations:** School of Biological Engineering, Dalian Polytechnic University, Dalian 116034, China

**Keywords:** lipoxins, anti-inflammatory mediators, biological activity, synthetic pathways, lipoxygenase

## Abstract

Lipoxins (LXs) have attracted widespread attention as a class of anti-inflammatory lipid mediators that are produced endogenously by the organism. LXs are arachidonic acid (ARA) derivatives that include four different structures: lipoxin A4 (LXA4), lipoxin B4 (LXB4), and the aspirin-induced differential isomers 15-epi-LXA4 and 15-epi-LXB4. Because of their unique biological activity of reducing inflammation in the body, LXs have great potential for neuroprotection, anti-inflammatory treatment of COVID-19, and other related diseases. The synthesis of LXs in vivo is achieved through the action of lipoxygenase (LO). As a kind of important enzyme, LO plays a major role in the physiological processes of living organisms in mammals and functions in some bacteria and fungi. This suggests new options for the synthesis of LXs in vitro. Meanwhile, there are other chemical and biochemical methods to synthesize LXs. In this review, the recent progress on physiological activity and synthetic pathways of LXs is summarized, and new insights into the synthesis of LXs in vitro are provided.

## 1. Introduction 

Inflammation is a physiological response of the organism to injury, and its adequate resolution is essential to restoring homeostasis. There is a set of precise inflammation regulation systems in organisms that dynamically regulate the anti-inflammatory and pro-inflammatory forces in vivo to maintain homeostasis balance. As a consequence, a range of immune responses function during the acute phase that ultimately prevent the transition into chronic inflammation [1,2]. This is operated by a metabolic switch that immune cells undergo at the peak of inflammation, whereby the cells recruited to the inflamed tissue stop producing inflammatory mediators and activate a pro-resolution program to biosynthesize the so-called specialized pro-resolving mediators (SPMs) [3,4]. SPMs derive from both omega-6 arachidonic acid and mostly omega-3 docosahexaenoic and eicosapentaenoic acids and include lipoxins (LXs), resolvins, maresins, and protectins, which together reduce the infiltration of leukocytes, induce the production of anti-inflammatory mediators, clear pathogens and debris, and promote tissue regeneration [5,6,7]. One of the hallmarks of unresolved inflammation is oxidative stress, which is defined as an imbalance between free radical production and the antioxidant system. It is associated with the presence of numerous oxidative products, such as reactive oxygen species (ROS) and reactive nitrogen species (RNS), which cause damage to human tissues through immune-mediated injury responses. When chronic inflammation occurs under this condition, SPMs also act as endogenous modulators of oxidative stress by reducing pro-oxidant mediators and concomitantly activating the anti-oxidant genes by means of their specific receptors [8,9].

LXs have attracted widespread attention as a class of anti-inflammatory lipid mediators that are produced endogenously by the organism. As bioactive trihydroxyeicosatetraenoic acids, LXs are usually synthesized through the metabolism of arachidonic acid (ARA) in invertebrates and mammals. The formation of LXs can be achieved by cells expressing lipoxygenase (LO), such as leukocytes (mainly polymorphonuclear neutrophils, PMN), platelets, endothelium, and epithelium, via intercellular interactions. The first biosynthetic route of LXs is the sequential oxygenation of ARA by 5-LO in PMN to produce leukotriene LTA4, then to form 5S, 6S, 15S-epoxytetraene by 15-LO in epithelium, and finally to yield LXs. In this route, ARA can also be first transformed to 15S-HPETE by 15-LO alternatively, then to 5S, 6S, 15S-epoxytetraene by 5-LO. The second route of LX biosynthesis involves 5-LO in PMN and 12-LO in platelets. LTA4, derived from ARA by the action of 5-LO, is converted to a delocalized cation by 12-LO and then forms LXs. The third route of LX biosynthesis involves the presence of aspirin, cyclooxygenase-2 (COX-2) in endothelium, and 5-LO in PMN, and therefore the end products are termed aspirin-triggered LXs [10,11,12].

It is known that the inflammatory reaction produces chemical mediators, including LXs, which in turn stimulate the resolution of inflammation. When LXs are produced in organisms, they exert potent biological activities to promote inflammation resolution. LXs act mainly through binding to their receptors. FPR2/ALX, a G protein-coupled receptor, is the predominant LX receptor. When inflammation occurs, a variety of peptides triggering pro-inflammatory signaling bind to the FPR2/ALX, and this affinity allows LXs or other mediators to act and can be up-regulated by a variety of cytokines, resulting in enhanced anti-inflammatory effects [13]. The expression level of the FPR2/ALX receptor influences the outcome of the inflammatory response [14].

To date, owing to the anti-inflammatory activities of LXs, it has promoted the exploration of the exogenous uptake of LX. In an attempt to examine the effects of LX dosage on resolution, it was found that the LX treatment of 10 μg/kg/day in rats reduced Gentamicin-induced kidney damage by inhibiting the up-regulated inflammatory mediators ICAM-1, TGFβ 1 protein levels, and TNF-α protein expression. At present, milligrams of commercially available synthetic LXs have been developed and used in some preclinical disease models. It is worth noting that studies have confirmed the anti-inflammatory effect of oral administration or injection of such pro-resolving mediators [15]. In this review, we summarized the recent progress on the biomedical efficacy, structure, synthetic pathway of LXs in vivo and in vitro, and microbial sources of LO, with a focus on the biomedical efficacy and synthesis of LXs in vitro. If the bottleneck in the synthesis of LXs in vitro can be broken, it is expected that LXs will play an important role in human immunity instead of antibiotics.

## 2. The Discovery of LXs

As early as 1984, Serhan identified a novel oxo derivative with a trihydroxytetraconjugated double bond structure from human leukocytes, which were LXs [16]. As an important endogenous anti-inflammatory lipid mediator in the body, LX acts mainly on G protein-coupled receptors to regulate the cellular response caused by inflammation, thus contributing to the timely subsidence of the inflammatory response.

Following the discovery of LXs, Schwab used high-throughput lipidomics to isolate ω-3 polyunsaturated fatty acid-derived pro-resolving mediators, resolvin and protectin, from mouse peritoneal inflammatory exudate, which formed the basis of the promoting subsidence molecules with LXs [5]. Together with LXs, they constituted the three emerging families of SPMs [6,17]. They achieved the effects of inflammation resolution by promoting cell apoptosis and reducing cell migration through phosphorylation. 

In recent years, many achievements have been made with respect to analogues of LXs [18,19,20]. Because of the rapid loss of activity of LXs caused by PG dehydrogenase-mediated oxidation and reduction after function in vivo, analogues of LXs were widely explored and studied [21]. Petasis synthesized the analogues of LXs in 2005 by chemical method and drew the conclusion that the triene core of LXs replaced by a more stable benzene ring resulted in metabolic stability while the analogues conserved biological activity, which provided new insight into the synthesis of analogues of LXs [18]. 

## 3. Structure of LXs

**Figure 1:**
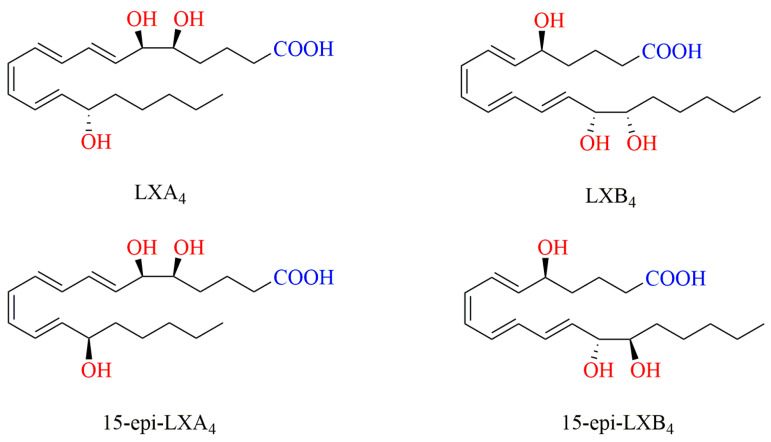
Structure of LXs.

Because of the difference in the conformation, the position of double bonds, and the formation of hydroxyl groups in the molecule, LXs can be classified into four types according to their biosynthetic pathways and the LOs involved in the process, i.e., LXA4, LXB4, and their 15-epimers of 15-epi-LXA4 and 15-epi-LXB4 (Figure 1). LXs have trihydroxyeicosatetraenoic acid in their structure. In addition, 15-epi-LXA4 and 15-epi-LXB4 are aspirin-induced LXs (aspirin-triggered lipoxins, ATLs), which have a more stable conformation and longer half-lives than LXA4 and LXB4 [13,22,23]. 

LXs generated in vivo are prone to rapid metabolic inactivation caused by monocytes, and research has shown that LX metabolites are biologically inactive [21]. Therefore, the inactivation of LXs makes it unfavorable to exert their biological activity. As a result, to reduce inactivation and extend the half-life of LXs in vivo, lots of LXs analogues have been chemically synthesized, and some of these analogues exhibit better stability and more potent bioactivity compared to the native LXs [20,24].

## 4. The Biomedical Efficacy of LXs

**Figure 2:**
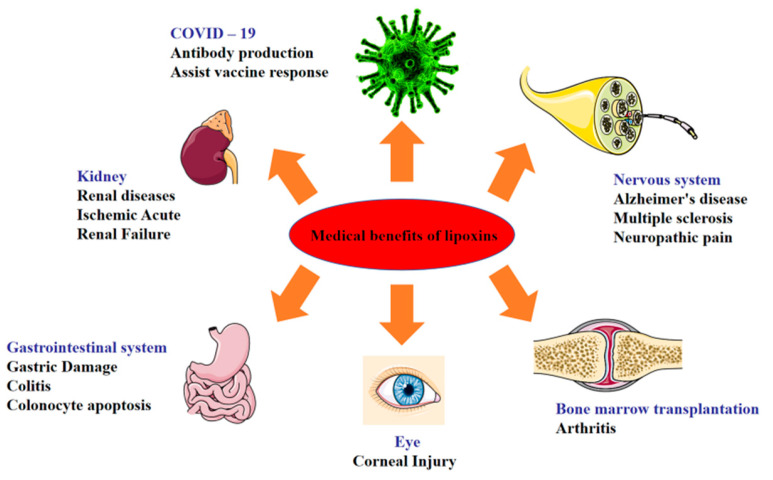
Medical benefits of LXs.

The function of LXs in reducing inflammation in the airway [25], cardiovascular [26], eye and kidney [27], brain [28], obesity [29], periodontal areas [30], gastrointestinal disorders, and liver has been reported. LXs play an important role in the immune regulation process of the body by regulating the immune response through a close connection with a variety of immune cells and immunomodulatory mediators [31]. They can inhibit the expression of pro-inflammatory factors, stimulate the expression of anti-inflammatory factors, inhibit the inflammatory effect of neutrophils, and promote the phagocytosis of apoptotic neutrophils by macrophages. They have good anti-inflammatory effects on the intestine, joints [32,33], lung [34], kidney [35], atherosclerosis [26,36], type II diabetes [37], and epidermal sites, and they also play a positive role in the treatment of COVID-19 as well as in neuroprotection (Figure 2).

### 4.1. Effects on COVID-19

When the virus of COVID-19 was exposed to cells, peroxisome proliferator-activated receptor (PPAR) could enhance the secondary antibody response of B cells, and the longevity of non-proliferating long-lived plasma cells located in the bone marrow increased, stimulated by LXB4. Additionally, it promoted the upregulation of BCL-2 family (a tripartite apoptosis control system consisting of a group of anti-apoptotic and two groups of pro-apoptotic proteins) factors. The above interactions could be useful for the secretion of intracellular antibodies, thus possibly assisting the vaccine response and preventing infection by COVID-19, which could also be used as a new idea for vaccine production [38,39,40,41].

### 4.2. Neuroprotection

In neuroinflammatory and neurodegenerative diseases such as multiple sclerosis, Alzheimer’s disease, and Parkinson’s disease, glial cell activation and high expression of inflammatory mediators within the central nervous system are key hallmarks, and SPMs can act as modulators of brain homeostasis and repair [42,43].

It has been found that there are different degrees of glial cell activation and high expression of inflammatory factors among the cerebral ischemic diseases Alzheimer’s disease and Parkinson’s disease. Some studies have suggested the mechanism by which LXA4 performs its function in neuroprotection. LXA4 alleviates neuroinflammation by regulating T cell responses and altering the lipid groups of the spinal cord, which causes strong anti-inflammatory and neuroprotective effects [44]. LXs exert significant anti-inflammatory and neuroprotective effects through the following aspects: (1) As agonists of peroxisome proliferator-activated receptor gamma (PPARγ), LXs can promote the synthesis of 5-LO and inhibit the synthesis of LTA4 by binding to PPARγ in cerebral ischemic diseases, and thus LXA4 synthesis is increased, exerting anti-inflammatory and neuroprotective effects [45]; (2) In rats with permanent middle cerebral artery occlusion (pMCAO), treatment of LX injection through the lateral ventricle significantly improves the symptoms of neurological deficits and reduces the percentage of cerebral infarct volume and neuronal cell death in rats [46,47,48]; and (3) The neuroprotective effect of LXs is closely related to the endogenous cannabinoid system [49,50,51].

### 4.3. Anti-Inflammatory

LXs can regulate the regression of inflammation by activating tissue regeneration in apoptotic neutrophil PMN [52]. In skin inflammation, LXA4 blocks the release of histamine during the interaction of mast cells with epithelial cells [53]. LXB4 inhibits T cell activation, the release of pro-inflammatory cytokines, and neutrophil chemotaxis, in addition to directly regulating mast cells and eosinophils [54,55]. The indirect mechanism for quickly eliminating allergic pulmonary inflammation by LXs may be due to inhibiting the activity of inflammatory mediators [56]. Moreover, LXA4 exists in dysregulation and disorders of lipid mediators in bronchitis and asthma lung ischaemia reperfusion injury (LIRI) in lung transplant patients. It was found that LXA4 treatment had anti-inflammatory, antioxidant, and endothelial protective effects on LIRI after lung transplantation in rats [57]. This finding provides therapeutic potential because of the efficacy of LXA4 in bronchiectasis. In conclusion, the experimental data adequately demonstrate several cellular mechanisms by which LXs regulate allergic airway inflammation and have a positive effect on the treatment of asthma disease.

The research on LXs’ effects on disease shows that LXA4 plays a major role in promoting the stability of atheromatous plaques, including reducing oxidative stress and necrosis of lesions and improving the cytosis of lesions, which is a clinical molecular signal for the formation of plaque [58]. Evidence for the efficacy of LXs as anti-arthritic therapeutics showed that LXs had the functions of dampening inflammation, promoting cartilage and bone repair, and having potent analgesic effects [59,60]. LXA4 also contributes to the induction of fibroblast MEF differentiation into brown adipocytes, which can reverse adipose tissue inflammation and improve insulin resistance and is considered a new drug target for obesity management [61]. Moreover, high levels of LXA4 are highly associated with low levels of type II diabetes incidence. By studying inflammatory changes in human beings and mice during parturition, Han and his colleagues found that epinephrine upregulates LOs expression and produces more LXA4, which acts as an anti-inflammatory agent in parturition [62].

LXs analogues have been used for the clinical treatment of diseases in recent years. A study focusing on molecular mechanisms showed that LXs analogues could enhance reprogramming and crosstalk between classical and non-classical innate immune cells, thus inducing the termination of the pro-inflammatory state and promoting the subsequent regression stage, which provided a possible treatment for inflammatory heart metabolic diseases [20]. In addition, research was performed for the asymmetric synthesis of four bicyclo-pentanes, including synthetic aromatic LXA4 mimetics, and the biological evaluation of analogues demonstrated the therapeutic potential of BCP-sLXms as a novel inflammatory regulator [63]. Moreover, it was found that the LXA4 analog BML-111 could reduce oxidative stress and glomerular podocyte injury by regulating the Nrf2 pathway [64]. These results indicated that LX analogues also had anti-inflammatory and therapeutic biological functions.

## 5. Biosynthetic Pathways of LXs In Vivo

**Figure 3:**
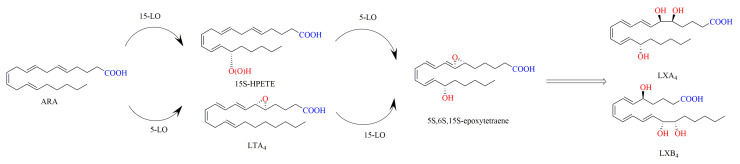
Synthesis of LXA4 and LXB4 in vivo by 15-LO/5-LO [12].

**Figure 4:**
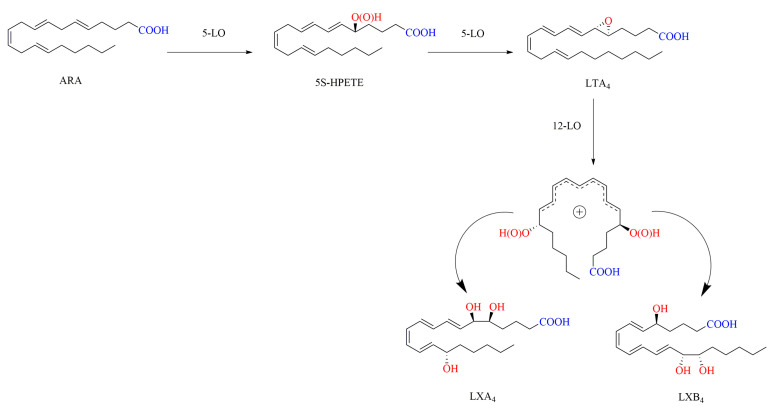
Synthesis of LXA4 and LXB4 in vivo by 5-LO/12-LO [12].

**Figure 5:**
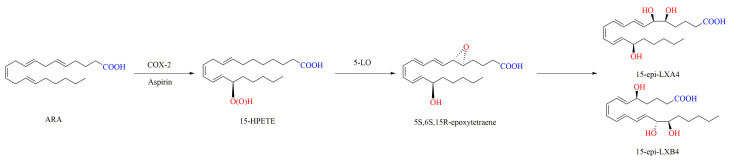
Synthesis of 15-epi-LXA4 and 15-epi-LXB4 in vivo by COX-2/5-LO induced by Aspirin [12].

In mammals, the generation of LXs derived from ARA is regulated by LOs [65,66]. During the biosynthesis pathways of LXs in vivo, the polyunsaturated fatty acid ARA is catalyzed for successive oxidation by LOs such as 5-LO, 15-LO, 12-LO, and COX-2. A total of three biosynthetic routes for LXs in vivo have been reported. In the 15-LO/5-LO route, ARA is oxygenated to 15S-HPETE by 15-LO and then transformed to the intermediate 5S,6S,15S-epoxytetraene by 5-LO, which is hydrolyzed to LXA4 and LXB4 by hydrolase. Alternatively, ARA can be first converted to LTA4 catalyzed by 5-LO, then to form 5S,6S,15S-epoxytetraene by 15-LO, and finally to yield LXA4 and LXB4 by hydrolase (Figure 3). In the 5-LO/12-LO route, 5-LO catalyzes the conversion of ARA to LTA4, and then 12-LO drives LTA4 to produce a delocalized cation, and finally this cation is attacked by water molecules either at C-6 to yield LXA4 or at C-14 to yield LXB4 (Figure 4). In the synthetic route of 15-epi-LXA4 and 15-epi-LXB4, COX-2 induced by Aspirin catalyzes the conversion of ARA to 15R-HPETE, which is then transformed by 5-LO to yield 5S,6S,15R-epoxytetraene, and finally is converted into 15-epi-LXA4 and 15-epi-LXB4 (Figure 5) [12,13,67,68]. 

## 6. Synthesis of LXs In Vitro 

### 6.1. Chemical Synthesis of LXA4 and LXB4 by Iodination Method

Back in 1985, Corey and his colleagues at Harvard University discovered a chemical synthesis method to obtain LXA4 and LXB4, and this method belonged to the category of iodization in essence [69,70,71]. In this method, LXA4 or LXB4 is chemically synthesized through hydroxyl protection of the staring compounds, changing the position of the double bonds, and introducing hydroxyl groups. The results indicated that LXA4 and LXB4 could be obtained in relatively high yields, but this method is very complicated to operate and more costly, which is not suitable for commercial production of LXs.

**Figure 6:**
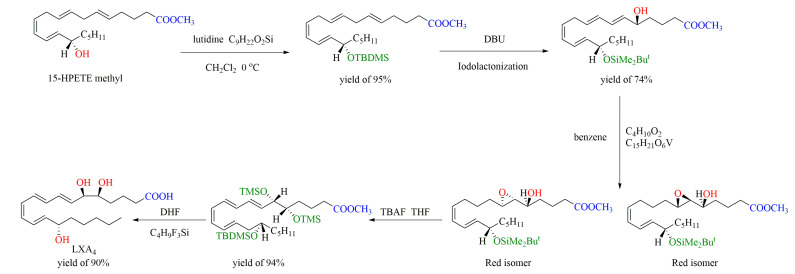
Chemical synthesis of LXA4 by iodization method (DBU: diazabicyclo-undecene; THF: Tetrahydrofuran; TBAF: Tetrabutylammonium fluoride hydrate; DHF: Dihydrofolic acid).

As shown in Figure 6, the chemical synthesis of LXA4 by the iodization method starts with 15S-HETE methyl ester, which first reacts with tert-butyl dimethylsilyl triflate and lutidine in methylene chloride at 0 °C to form tert-butyldimethylsilyl ether. The resulting tert-butyldimethylsilyl ether was then converted into 5-hydroxy-6E-silyl ether-methyl ester by the standard iodolactonization method consisting of sequential reactions, and finally the compound LXA4 was formed, which was determined by reversed-phase HPLC analysis [70]. 

**Figure 7:**
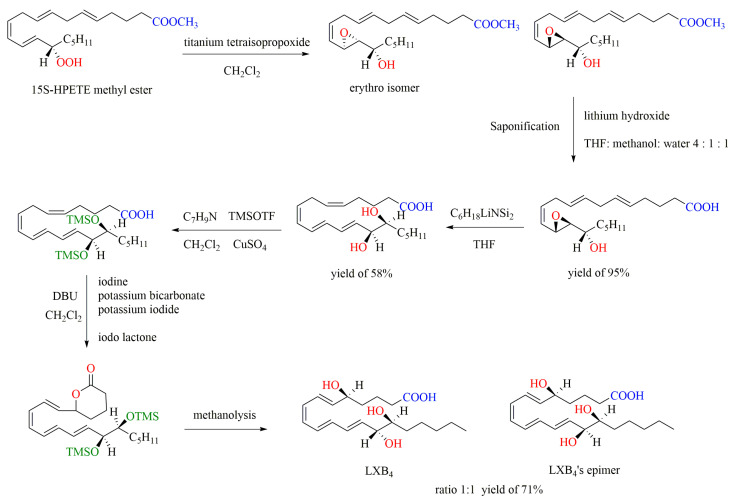
Chemical synthesis of LXB4 by iodization method (THF: Tetrahydrofuran; TMSOTF: Trimethylsilyl trifluoromethanesulfonate; DBU: diazabicyclo-undecene).

As shown in Figure 7, the chemical synthesis of LXB4 by the iodization method starts with 15S-HPETE methyl ester, which first reacts with titanium tetraisopropoxide in methylene chloride to form threo epoxy alcohol and the erythro isomer in a ratio of 3:1. Sanonification of threo epoxy alcohol with lithium hydroxide in THF–methanol–water and acidification afford hydroxy epoxide, which undergoes sequential reactions and finally gives LXB4 and its isomer [69].

### 6.2. Enzymatic Synthesis of LXA4 and LXB4 Catalyzed by LO from Soybean

Sok and colleagues used LO derived from soybean to convert ARA into LXA4 and LXB4. ARA dissolved in ammonium hydroxide was suspended in 0.1 mol/L sodium borate buffer (pH 8.7) and cooled to 4 °C. The reaction was initiated by the addition of LO from soybeans and incubated at 4 °C for 2 h and then at 20 °C for 1 h. Separately, 5,15-dihydroperoxyeicosatetraenoic acid was incubated with soybean LO in 0.1 mol/L sodium borate buffer (pH 8.7) at 20 °C. The reaction was stopped by adjusting the pH to 4.0, and the reaction mixture was extracted with ethyl ester. The extract was subjected to a purification procedure. Based on the chromatographic and spectrometric analysis, the products were identified as LXA4 and LXB4. The results indicated that LO from soybean could catalyze the formation of LXA4 and LXB4 from ARA. Meanwhile, the exposure of 5,15-dihydroperoxyeicosatrienoic acid to soybean LO also produced identical products of LXA4 and LXB4, indicating that 5,15-dihydroperoxyeicosatetraenoic acid served as an intermediate during the conversion process of ARA into LXA4 and LXB4 by soybean LO. However, the total yield of LXA4 and LXB4 was very low, only ranging between 1% and 2% [72].

### 6.3. Chemical-Enzymatic Synthesis of LXA4 and LXB4 

**Figure 8:**
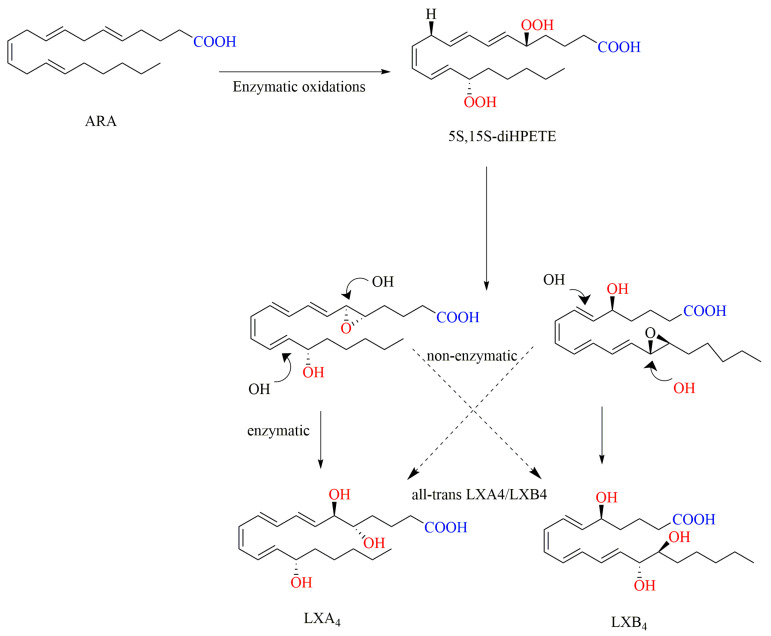
Chemical-enzymatic synthesis of LXA4 and LXB4 from ARA [73].

Adams et al. reported the enantiospecific and stereospecific synthesis of LXA4 and LXB4 [73]. In this method, both chemical and enzymatic steps were used to convert leukotriene LTA4 and its unnatural epoxy isomers into four diastereomeric 5(S),6(S),l5(S)-trihydroxy-7,9,13-trans-11-cis-eicosatetraenoic acids. As shown in Figure 8, the sequential enzymatic oxidation of ARA at C-15 and C-5 catalyzed by LO results in the formation of 5S,15S-diHPETE. Subsequently, through stereospecific enzymatic dehydration, 5S,6S-epoxide along with 7,9,13-trans-11-cis geometry is generated. The resulting epoxide undergoes enzymatic hydrolysis in biological reactions or non-enzymatic nucleophilic addition with water in chemical reactions, yielding LXA4 and LXB4, respectively.

## 7. Bottleneck in the Synthesis of LXs In Vitro

The synthesis of LXs in vitro through the three above-mentioned routes still faces challenges. For the chemical synthesis of LXA4 and LXB4 by the iodination method, the economic viability and the process complexity of the route should be taken into account [69,70]. For the chemical-enzymatic synthesis of LXs from ARA, the purity of trans LXs needs to be increased, and in the meantime, they may differ from natural LXs in terms of biological activity [72]. The bottlenecks in the synthesis of LXs in vitro need to be broken. Compared with chemical synthesis, biological synthesis of LXs has great potential for development, where the catalyst LO is indispensable.

Studies have found that LO exists widely in organisms such as plants, animals, algae, fungi, yeast, and bacteria [74,75,76,77,78,79]. In plants, LO primarily reacts with linoleic acid and linolenic acid. In animals, LO mainly reacts on ARA [70,80], converting it into biologically active lipid mediators, including LTA4 or LXs, which play a crucial role in inflammatory responses [81,82]. To realize effective enzymatic synthesis of LXs in vitro, it is crucial to obtain suitable LO by means of gene engineering or protein engineering.

### 7.1. Microbial Sources of LO

#### 7.1.1. Bacterial Sources

Bacterial sources of LO have been extensively investigated. The genome of the diazotrophic *cyanobacterium Cyanothece * sp. ATCC 51142 contains two genes homologous to LOs. One of them, the *Csplox2* gene, is cloned in *E. coli*, and the protein is expressed and purified. The purified enzyme has a molecular weight of 65 kDa and belongs to a prokaryotic mini-LO, which mainly catalyzes the conversion of linoleic acid, or ARA, into LX precursors such as 11-HETE, 13-HETE, and 15-HETE [81]. 

DNA isolated from the injured strain of *Nostoc punctiforme strain* PCC-73102 has at least two different LO sequences [82]. They have been cloned as cDNAs named *NpLOX1* and *NpLOX2*. Both proteins were identified as linoleic acid 13-LO by expressing in *E. coli*. *NpLOX1* was characterized by a maximum pH value of 8.0 and a preferred substrate of α-linolenic acid. Injured bacterial extracts contained more 13-LO-derived hydroperoxides compared to uninjured cells, and the non-esterified lipids were 30-fold higher than the esterified lipoproteins. The results suggest that 13-LO acting on free fatty acids predominates in *N. punctiforme strain* PCC-73102 when injured.

*Pseudomonas aeruginosa* is an opportunistic pathogen that can cause hospital and chronic infections in immunocompromised patients [83]. Analysis of the whole genome sequence of the pathogen *P. aeruginosa* revealed an ORF (PA1169) with high homology to LOs. Using liquid chromatography-mass spectrometry (LC-UV-MS-MS), PA1169 was shown to encode a bacterial lipoxygenase (LoxA), which converts ARA into 15-hydroxyeicosatetraenoic acid (15-HETE), a precursor for the synthesis of LXs. Through tree graph analysis, LoxA is not significantly orthologous to a specific plant or animal Los. Compared to other Los, the LoxA protein does not seem to contain transmembrane fragments. Moreover, the signal peptide composed of the first 19 amino acids of the protein was predicted, and it was found that the protein might be secreted. Los secreted by *P. aeruginosa* can regulate mechanisms of host defense and inflammation by altering the biosynthesis of local chemical mediators. Due to the highly conservative nature of the *LoxA* gene, it seems not to be easily reproduced in vitro. The *LoxA* gene is well conserved; it seems likely to be expressed in situ in specific scenarios, but it seems not to be easily recapitulated in vitro. 

It is worth mentioning that bacterial LOs have been reported to be active against a broad range of polyunsaturated fatty acids, such as linoleic acid, α-linolenic acid, γ-linolenic acid, ARA, eicosapentaenoic acid (EPA), and docosahexaenoic acid (DHA) [84,85,86,87]. And the substrate preference of bacterial LOs was different. The enzymes from *B. thailandensis* E264, *Rivularia * sp. PCC-7116, *Calothrix * sp. HK-06, *Tolypothrix bouteillei, Sphingopyxis macrogoltabida,* and *Pseudomonas * sp. 42A2 were reported to have the highest activity towards linoleic acid [84,87,88]. Los from *Archangium violaceum* and *Endozoicomonas numazuensis* displayed the highest activity towards ARA [85,89]. LO from *P. aeruginosa* PAO1 showed the highest activity towards γ-linolenic acid, followed by DHA [90]. LOs from *M. fulvus* were reported to be most active with EPA [91]. Among all the characterized bacterial LOs, those from *Rivularia* sp. PCC-7116 and *Calothrix* sp. HK-06 have the highest activities reported so far [87]. The wide diversity in substrate preference of bacterial LOs raises the opportunity to act on different substrates. As a result, LOs of bacterial origin have a better and wider range of applications.

#### 7.1.2. Fungal Sources

There are very few reports on LO from fungal sources. Interestingly, fatty acid-derived aldehydes, ketones, and alcohols were found to be released in moss, *Physcomitrella patens,* after being injured. The researchers isolated a related enzyme, LO, that was similar to the structure of the plant type-2 LO domain through sequence analysis. The gene was introduced into *E. coli* for cloning and expression, and the enzyme activity was analyzed by liquid chromatography. It was found that ARA and EPA were the preferred substrates, and they might be involved in the formation of signaling or defense molecules in an efficient manner, thus contributing to the broad resistance of mosses to pathogens [92]. However, the work did not describe the activity and stability of LO with quantitative means.

## 8. Conclusions

Since the discovery of LXs and other SPM mediators, many studies have shown their relevant biomedical functions, such as potent anti-inflammatory effects. Therefore, LXs have become candidates to fight against some diseases with great development prospects. However, currently, LXs are limited to biosynthesis in physiological environments and at disease levels, and there seem to be challenges in the synthesis of LXs in vitro using chemical or biochemical methods. Is it possible to use gene engineering to express LO in model microorganism hosts and achieve the synthesis of LXs? Considering the bacterial sources of LOs at present, we believe that this method is feasible and could potentially solve the problem of using lipoxins as a substitute for current anti-inflammatory drugs.

## Data Availability

Data will be made available on request.
